# SARS-CoV-2 Molecular Epidemiology Can Be Enhanced by Occupational Health: The Experience of Monitoring Variants of Concern in Workplaces in Rio de Janeiro, Brazil

**DOI:** 10.3389/fmed.2022.862284

**Published:** 2022-04-29

**Authors:** Sergio N. Kuriyama, Bruna Farjun, Bianca Monteiro Henriques-Santos, Adriana Cabanelas, Juliana Lourenço Abrantes, João Gesto, Antonio A. Fidalgo-Neto, Thiago Moreno L. Souza

**Affiliations:** ^1^SESI Innovation Center for Occupational Health, Rio de Janeiro, Brazil; ^2^SENAI Innovation Institute for Green Chemistry, Rio de Janeiro, Brazil; ^3^Instituto de Ciências Biomédicas, Universidade Federal do Rio de Janeiro, Rio de Janeiro, Brazil; ^4^Laboratório de Imunofarmacologia, Instituto Oswaldo Cruz, Fundação Oswaldo Cruz, Rio de Janeiro, Brazil; ^5^National Institute for Science and Technology for Innovation on Diseases of Neglected Populations (INCT/IDPN), Center for Technological Development in Health (CDTS), Oswaldo Cruz Foundation (Fiocruz), Rio de Janeiro, Brazil

**Keywords:** COVID, SARS-CoV-2, occupational medical care, surveillance, variants of concern (VOCs), molecular sequence, next generation (deep) sequencing (NGS)

## Abstract

The emergence of the severe acute respiratory syndrome coronavirus 2 (SARS-CoV-2) has led to extra caution in workplaces to avoid the coronavirus disease 2019 (COVID-19). In the occupational environment, SARS-CoV-2 testing is a powerful approach in providing valuable information to detect, monitor, and mitigate the spread of the virus and preserve productivity. Here a centralized Occupational Health Center provided molecular diagnosis and genomic sequences for companies and industries in Rio de Janeiro, Brazil. From May to August 2021, around 20% of the SARS-CoV-2 positive nasopharyngeal swabs from routinely tested workers were sequenced and reproduced the replacement of Gamma with Delta variant observed in regular surveillance programs. Moreover, as a proof-of-concept on the sensibility of the occupational health genomic surveillance program described here, it was also found: i) the primo-identification of B.1.139 and A.2.5 viral genomes in Brazil and ii) an improved dating of Delta VoC evolution, by identifying earlier cases associated with AY-related genomes. We interpret that SARS-CoV-2 molecular testing of workers, independent of symptom presentation, provides an earlier opportunity to identify variants. Thus, considering the continuous monitoring of SARS-CoV-2 in workplaces, positive samples from occupation health programs should be regarded as essential to improve the knowledge on virus genetic diversity and VoC emergence.

## Introduction

The emergence of the severe acute respiratory syndrome coronavirus 2 (SARS-CoV-2), the causative agent of 2019 coronavirus disease (COVID-19), has led to over 200,000 death/month globally in approximately 2 years (1). Deaths, hospitalizations, long-term sequela, and work absence are among the COVID-19-associated factors that have led to economic depression (2). Especially in low- and middle-income countries, such as Africa and Latin America, where budget constraints are constant and reinforced under economic uncertainties, science, and public health funding may become at risk. Indeed, the percentage of sequenced SARS-CoV-2 genomes among confirmed cases is lower in these countries than in wealthier nations (3, 4).

SARS-CoV-2 surveillance has been built on influenza monitoring networks (5). In brief, State laboratories share samples from syndromic patients (under different levels of assistance, from immediate attention to deceased patients) with National Influenza Centers (NIC), which subsequently consolidate with World Health Organization (WHO) Collaborating Centers (CC) the variants to be prioritized for regular vaccine updates and pandemic preparedness. However, besides this standard surveillance component, SARS-CoV-2 has led to extra caution in workplaces to avoid COVID-19 (6), differently than any other emerging pathogens. In the occupational environment, SARS-CoV-2 testing is a powerful approach in providing valuable information to decision-makers to adopt measures to mitigate the spread of the virus, preserving a healthy workplace and productivity.

Thus, SARS-CoV-2 pandemics imposed a necessity that occupational health programs adapt to organize and implement the early identification of workers with SARS-CoV-2 positive diagnosis (6) and even vaccine advocacy. Nevertheless, the natural history of COVID-19 is challenging because asymptomatic and pre-symptomatic patients may impose a risk of virus spread among co-workers (7). In companies with an implemented occupational health system, routine systematic testing of workers, regardless of their symptoms, could provide recommendations for self-quarantine, avoiding SARS-CoV-2 spread, and better monitoring the clinical evolution of COVID-19.

As SARS-CoV-2 spilled over in the wet market in Wuhan, China, workers from there were among the very first patients with COVID-19, including the most likely patient “zero” (8). Apparently, in this market, two independent episodes posed the risk of SARS-CoV-2 spillover from animals to human (9), reinforcing the notion that this workplace was endowed with substantial risk for the emergence of new viruses. Although ‘pneumonia of unknown origin' was the diagnosis assigned to the initial patients who underwent hospitalization, COVID-19 hospitalization was necessary in 5–7% of the initial cases, meaning that occupational health surveillance in Wuhan's wet market could have identified SARS-CoV-2 among the other 95–93% of persons with asymptomatic or mild symptoms (8) and allowed earlier contingency of the very first infected individuals. Although the wet market was most likely the epicenter of the virus spillover, other workplaces with lower risks of virus emergence also contribute to SARS-CoV-2 chains of transmission (10, 11).

SARS-CoV-2 surveillance must be a priority to properly represent viral genetic diversity within a community. Beyond regular surveillance, occupational health can readily provide opportunities for early detection. SARS-CoV-2 pathogenesis and ability to escape the humoral immune response may vary among the variants of concern (VoC) (12), making it necessary to effectively and efficiently identify COVID-19 clusters to categorize the viral lineages. Indeed, the Gamma, Delta, and Omicron VoCs emerged from uncertain origins (13–15). Thus, every opportunity to catalog SARS-CoV-2 genetic diversity should be taken to fulfill the phylogenetical and epidemiological puzzle imposed by this virus. Among these opportunities, the integration of occupational health and SARS-CoV-2 surveillance has been proposed (16). Still, it has never been tested whether programs in workplaces have the sensitivity to detect variants and lineages with similar trends as regular “influenza-like” surveillance programs.

In Rio de Janeiro, Brazil, centralized infrastructures support social and health programs for industries and other entrepreneurial activities (National Industrial Apprenticeship Service, SENAI; and Industry Social Service, SESI). From September 2020 to May 2021, the SESI Innovation Center for Occupational Health has supported industrial and service companies in this State to implement and perform the detection of SARS-CoV-2 through RT-PCR (17). From May 2021 forward, SARS-CoV-2 genome sequencing was implemented to reinforce the molecular surveillance associated with occupational health. Therefore, we evaluated the SARS-CoV-2 genetic diversity in workers from Rio de Janeiro and correlated these findings to those from regular surveillance programs.

## Materials and Methods

### Population and Ethics

The mass testing program for COVID-19, proposed by SESI Innovation Center for Occupational Health (FIRJAN, Rio de Janeiro—Brazil), screened workers from industry service companies in Rio de Janeiro, Brazil, independently of any symptoms through RT-qPCR in nasopharyngeal swab specimens. Positive samples were randomly selected for genomic analyses through deep sequencing from May to August 2021.

The National Committee approved the present study of Research Ethics and the Ethics Committee of Hospital Universitário Clementino Fraga Filho under protocol number 4,317,270.

### RNA Extraction

For the mass testing program, RNA was extracted from nasopharyngeal swabs transported in DMEM using the Total RNA Purification Kit (Ref.: 400793, Agilent Technologies) with the Bravo Automated Liquid Handling Platform (Agilent Technologies), according to the manufacturer's protocols.

For the sequencing analyses, we performed a new total viral RNA extraction from stored frozen stock using ReliaPrep™ Viral TNA Miniprep System (Ref #AX4820, Promega) following manufacturers' protocols with minor modifications. Briefly, we used 1,000 μl of the inoculated medium and adjusted proteinase K, cell lysis buffer, and isopropanol volumes accordingly.

### RT-qPCR

RNA samples were screened through RT-qPCR following CDC protocols for SARS-CoV-2 detection, using viral targets N1 and N2, and the human gene for RNase P, using a primer-probe kit from IDT (Ref #10006713) and TaqPath™ 1-Step RT-qPCR Master Mix (Ref.: A15300, Applied Biosystems), following manufacturers' protocols for reaction volumes and cycling conditions (18). We considered positive samples those detectable for the three targets simultaneously with a cycle threshold value (CT) below 40.

### SARS-CoV-2 Sequencing, Processing, and Analysis

RNA from the positive nasopharyngeal swabs with the highest viral loads (ct values below 30) were randomly chosen for sequencing. SARS-CoV-2-related reads were enriched with Atoplex (version 1.0; MGI Tech Co., China) and sequenced by 100-nt pair-ends DNA nanoball technology on a DNBSEQ-G50 apparatus (MGI Tech Co.) (19). Consensus FASTA genomes were generated by the GenomeDetective (20) (https://www.genomedetective.com/) online platform from raw sequencing data. Consensus genomes were aligned and assigned to pangolin lineages with the phylogenetic assignment of outbreak lineages (Pangolin, Galaxy Version 3.1.17+galaxy1) (21). Aligned FASTA files were also assigned to variants and quality checked by NextClade (https://clades.nextstrain.org/, version 1.10.0) (22). The output JSON file from NextClade was used to generate phylogeny through https://auspice.us/ (version 0.8.0) (23, 24).

### Statistical Analysis

Standard descriptive statistics were used to describe the study population. Continuous variables were reported as appropriate as the mean ± standard deviation or median (range). Comparative analyses were performed using OpenEpi software (25). Statistical significance was reached when *p* < 0.05.

## Results

### Clinical-Demographical Characteristics of the Occupational Health Cohort

In Rio de Janeiro, Brazil, we detected 292 positive cases of SARS-CoV-2 from May to August 2021, a period when the Delta variant was introduced in Brazil (26). Among these cases, 72 individuals presented samples with the highest virus loads, with CT values up to 29, prioritized for next-generation sequencing. We obtained 50 high-quality, full-length consensus genomes, with quality scores above 30 for base calling, at least 10x of depth, and adequate NextClade assignment. The distribution of the sequenced SARS-CoV-2 cases among economic activities is representative of the workers who tested positive during this period (Table 1). Interestingly, the occupational health cohort was younger than those patients assisted at regular health units (Table 2) (26, 27), consistent with an economically active population. Patients tended to be male, and no severe cases were observed in our cohort (Table 2).

**Table 1:** The distribution of tested workers according to the industrial sector.

**Industrial and entrepreneurial sectors**	**Subset subjected sequencing**	**Total positive cases**
Services (health, realtor, informatics, administrative, commerce)	48%	36%
Construction	4%	7%
Processing industries	31%	37%
Extractive industries	17%	20%

*The chi-square p-value for this table is 0.34693 and, therefore, not statistically significant at the p < 0.05 level*.

**Table 2:** Characteristics of the occupational health cohort.

	**Occupational health cohort**	Regular surveillance in Rio de Janeiro^^*^^
Age (years-old, median-IQR)	38 ± 11	63 ± 20
Males (%)	56	52
Mortality (%)	0	4.2
Hospitalization and other reported medical complication (%)	0	7.1

* *Data obtained from regular surveillance bulletins (26, 27)*.

### SARS-CoV-2 Variants in the Workers

**Figure 1:**
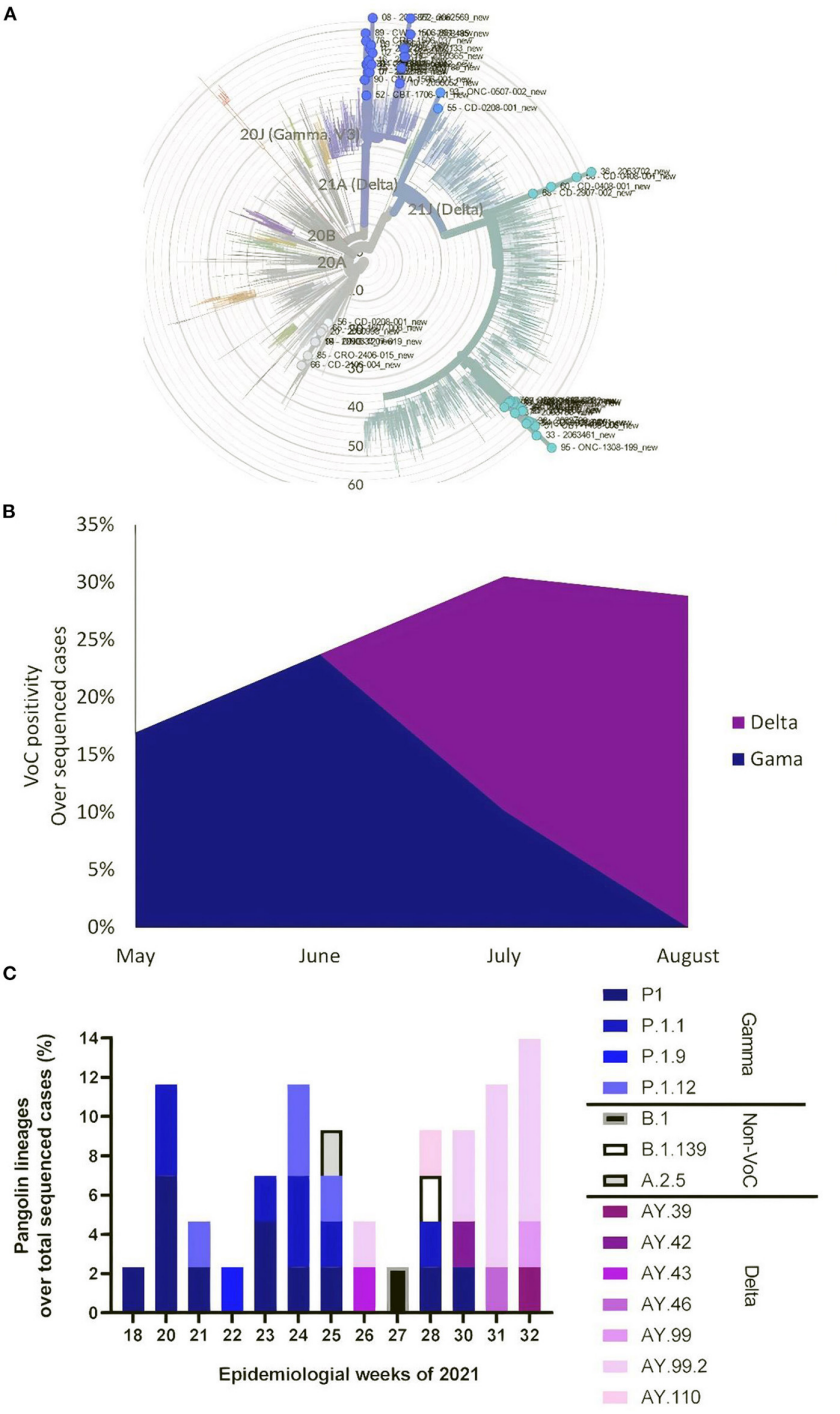
Occupational health-associated SARS-CoV-2 molecular surveillance. Of the 292 positive nasopharyngeal swabs from a cohort of workers in Rio de Janeiro, Brazil collected between May and August, 72 samples were randomly chosen for SARS-CoV-2 sequencing (Atoplex version 1.0, using the pair-end of 100 nucleotides in the DNBSEQ-G50 sequencer). Fifty full-length consensus genomes were generated with10x depth and quality scores > 30. **(A)** Maximum-likelihood phylogeny showing mutation tree compared with 2265 genomes from Supplementary File 1. **(B)** Distribution of the Gamma and Delta variants, assigned by NextClade version 1.10.0, per month. **(C)** Distribution of Pangolin lineages, assigned by Phylogenetic Assignment of Outbreak Lineages (Galaxy Version 3.1.17 + galaxy1) per epidemiological week. The genomes generated in this study are deposited in GenBank under accession codes: OM188304-OM188353.

The full-length SARS-CoV-2 genomes belonged to the Gamma and Delta clades (Figure 1A). The Delta variant started to replace Gamma from June to August (Figure 1B), consistent with public data on Brazilian molecular surveillance for SARS-CoV-2 from health care units (http://www.genomahcov.fiocruz.br/dashboard/). The distribution of the Gamma and Delta SARS-CoV-2 lineages per epidemiological week (Figure 1C) also indicates the timely identification of each variant (http://www.genomahcov.fiocruz.br/dashboard/) in the occupation health cohort.

We identified P.1 and P.1-related lineages in late May, consistent with virus circulation in Rio de Janeiro (http://www.genomahcov.fiocruz.br/dashboard/). The genomes added by this study increased the genetic diversity of the P.1-related lineages in Brazil and demonstrated the early identification of P.1.1, P1.1.9, and P.1.12 genomes from occupational health samples (Figure 1C) (https://outbreak.info/situation-reports/gamma?loc=BRA&loc=USA&loc=JPN&selected=BRA).

Similar to the Brazilian molecular surveillance program (Dashboard—Genomahcov—Fiocruz), we identified Delta AY-related lineages during late June/early July (Figure 1C). Through this study, we increased around 20% the number of high-quality genomes from this period in Rio de Janeiro, Brazil, and, more importantly, found that AY.42, AY.43, AY.46, AY.99, and AY.110 linages occurred contemporaneous or even a few weeks earlier than previously described (https://outbreak.info/situation-reports/delta?loc=IND&loc=GBR&loc=BRA&selected).

Occupational health samples also provide an opportunity to identify USA-related lineages, B.1.139 and A.2.5 (Figure 1C), not previously reported in Brazil (https://cov-lineages.org/lineage.html?lineage=B.1.139, https://cov-lineages.org/lineage.html?lineage=A.2.5).

Our results reinforce that molecular surveillance of infectious diseases could be enhanced by integrating occupational health initiatives that routinely monitor workers. For instance, during the COVID-19 pandemic and the emergence of SARS-CoV-2 variants, these samples could represent additional opportunities to observe viral genetic diversity.

## Discussion

Brazil and other Latin American countries struggle to combat COVID-19, including limitations to access diagnosis, intensive care units, and vaccines. Consequently, overcrowded megalopoleis, such as the City of Rio de Janeiro, have a case-fatality ratio of 7.1%, almost two times higher than its State and Country (26, 27). If the City or State of Rio de Janeiro were a country, it would fit among the three regions with the highest death rates in the world (1). Nevertheless, the percentage of sequenced SARS-CoV-2 genomes per confirmed case is low in Brazil, 0.3 genomes per 1,000 confirmed cases (4, 28). Given that Brazil has more representative sequences deposited in GISAID than any other South American country, other Latin American countries struggle to catalog the SARS-CoV-2 genetic diversity (4, 28). In Brazilian studies analyzing circulating SARS-CoV-2 variants using health surveillance programs, only a small subset of samples, around 1%, were sequenced (14, 29). In low- and middle-income countries, which are overwhelmed by the COVID-19 pandemic, most of the resources in public health are consumed by patient assistance, leaving a limited budget for disease prevention and prediction through continuous surveillance.

COVID-19 severely impacted Brazil (1), and, despite the limitation of cataloging only a subset of virus genomes, the pangolin lineages B.1.1.28 and B.1.1.33 are the most representative (Dashboard—Genomahcov—Fiocruz). The Gamma VoC also evolved from the B.1.1.28 lineage (14). The introduction of B.1.1.28 and B.1.1.33 likely occurred early in 2020, which overlaps with the Brazilian Carnival (30, 31), when no specific lockdown or self-quarantine measures were implemented. Stochastic events are thus not only associated with SARS-CoV-2 spillover from animal to humans (8) but also with virus dissemination. The SARS-CoV-2 spillover was associated with occupational risk for the workers at the wet market in Wuhan (8). When surveillance programs based on symptomatic patients identified the first cases of SARS-CoV-2, over 90% of asymptomatic or mildly affected individuals were likely circulating (8). Therefore, routine testing becomes an important tool to communicate public health surveillance systems early.

Our data points out that companies with implemented occupation health surveillance for COVID-19 may have an adequate and timely opportunity to increase awareness of the genetic diversity of circulating strains of SARS-CoV-2. Indeed, it has been proposed that occupational health should be an integral component of the response to COVID-19 (6, 16). Still, it is here that we document the experience from Rio de Janeiro, where a centralized Center for Occupational Health has reduced the dependence on governmental funding to catalog SARS-CoV-2 genetic diversity and find VoC with similar trends to a regular surveillance system. By sequencing representative samples, around 20%, from a cohort with characteristics than those on regular SARS-CoV-2 genomic surveillance networks, we even found: i) the primo-identification of the B.1.139 and A.2.5 viral genomes in Brazil; and ii) an improved dating of Delta VoC evolution, by identifying earlier cases of associated with AY-related genomes. We interpret that SARS-CoV-2 molecular testing of workers, independently of symptoms, has allowed an earlier opportunity to identify variants than regular health surveillance programs. These regular programs are primarily dependent on the spontaneous demand of syndromic patients and the cataloging of viral genetic diversity from hospitalized and deceased individuals, which may take weeks after the onset of illness to be identified.

## Conclusion

With the emergence of variants that escape the humoral immune response (12), companies must continuously monitor their workers for SARS-CoV-2. Under the auspicious of this investigation, we interpret that SARS-CoV-2-positive samples from occupation health programs should be considered as necessary as those from regular health surveillance programs to expand our awareness knowledge on virus genetic diversity and VoC emergence. We propose continuously performing occupational health surveillance to screen for other communicable diseases and identify possible chains of transmission.

## Data Availability Statement

The datasets presented in this study can be found in online repositories. The names of the repository/repositories and accession number(s) can be found in the article in the legend of Figure 1 and the Supplementary Material.

## Ethics Statement

The studies involving human participants were reviewed and approved by National Committee of Research Ethics and by the Ethics Committee of Hospital Universitário Clementino Fraga Filho under protocol number 4,317,270. The patients/participants provided their written informed consent to participate in this study.

## Author Contributions

SK and AF-N coordinated the cohort. BF, BM, AC, and JG performed the sequencing. JA, JG, and TS analyzed the data. SK, AF-N, and TS conceptualized the study. All authors prepared the manuscript.

## Funding

Funding was provided by Industry Federation of Rio de Janeiro (FIRJAN), Conselho Nacional de Desenvolvimento Científico e Tecnológico (CNPq), Fundação de Amparo à Pesquisa do Estado do Rio de Janeiro (FAPERJ) and Coordenação de Aperfeiçoamento de Pessoal de Nível Superior-Brasil (CAPES)-Finance Code 001. CNPq, CAPES and FAPERJ also support the National Institutes of Science and Technology Program (INCT-IDPN, 465313/2014-0).

## Conflict of Interest

The authors declare that the research was conducted in the absence of any commercial or financial relationships that could be construed as a potential conflict of interest.

## Publisher's Note

All claims expressed in this article are solely those of the authors and do not necessarily represent those of their affiliated organizations, or those of the publisher, the editors and the reviewers. Any product that may be evaluated in this article, or claim that may be made by its manufacturer, is not guaranteed or endorsed by the publisher.

## References

[1] Dong E Du H Gardner L. An interactive web-based dashboard to track COVID-19 in real time. Lancet Infect Dis. (2020) 20:533–4. 10.1016/S1473-3099(20)30120-1 32087114 PMC7159018

[2] Shrestha N Shad MY Ulvi O Khan MH Karamehic-Muratovic A Nguyen U-SDT. The impact of COVID-19 on globalization. One Health. (2020) 11:100180. 10.1016/j.onehlt.2020.100180 33072836 PMC7553059

[3] Oude Munnink BB Worp N Nieuwenhuijse DF Sikkema RS Haagmans B Fouchier RAM. The next phase of SARS-CoV-2 surveillance: real-time molecular epidemiology. Nat Med. (2021) 27:1518–24. 10.1038/s41591-021-01472-w 34504335

[4] Brito AF Semenova E Dudas G Hassler GW Kalinich CC Kraemer MUG. Global disparities in SARS-CoV-2 genomic surveillance. medRxiv. (2021) 2021.08.21.21262393. 10.1101/2021.08.21.21262393 34462754 PMC8404891

[5] WHO. World Health Organization. (2011). Available online at: https://web.archive.org/web/20111003070329/http://www.who.int/influenza/gisrs_laboratory/en/ (accessed January 19, 2022).

[6] Baker MG. Occupational health surveillance as a tool for COVID-19 prevention. Am J Public Health. (2021) 111:999–1001. 10.2105/AJPH.2021.306269 33950719 PMC8101572

[7] Qiu X Nergiz AI Maraolo AE Bogoch II Low N Cevik M. The role of asymptomatic and pre-symptomatic infection in SARS-CoV-2 transmission-a living systematic review. Clin Microbiol Infect. (2021) 27:511–9. 10.1016/j.cmi.2021.01.011 33484843 PMC7825872

[8] Worobey M. Dissecting the early COVID-19 cases in Wuhan. Science. (2021) 374:1202–4. 10.1126/science.abm4454 34793199

[9] Gao G Liu W Liu P Lei W Jia Z He X. Surveillance of SARS-CoV-2 in the environment and animal samples of the Huanan Seafood Market. Res. Squ. (2022). 10.21203/rs.3.rs-1370392/v1

[10] Liu P Yang M Zhao X Guo Y Wang L Zhang J. Cold-chain transportation in the frozen food industry may have caused a recurrence of COVID-19 cases in destination: successful isolation of SARS-CoV-2 virus from the imported frozen cod package surface. Biosafety Health. (2020) 2:199–201. 10.1016/j.bsheal.2020.11.003 33235990 PMC7676848

[11] Lehnertz NB Wang X Garfin J Taylor J Zipprich J VonBank B. Transmission dynamics of severe acute respiratory syndrome coronavirus 2 in high-density settings, Minnesota, USA, March–June 2020. Emerg Infect Dis. 27, 2052–2063. 10.3201/eid2708.204838 34138695 PMC8314815

[12] Bastos LS Ranzani OT Souza TML Hamacher S Bozza FA. COVID-19 hospital admissions: Brazil's first and second waves compared. Lancet Respir Med. (2021) 9:e82–e83. 10.1016/S2213-2600(21)00287-3 34273268 PMC8279962

[13] Where did ‘weird' Omicron come from? Available online at: https://www.science.org/content/article/where-did-weird-omicron-come (accessed January 18, 2022).

[14] Naveca FG Nascimento V de Souza VC Corado A de L Nascimento F Silva G. COVID-19 in Amazonas, Brazil, was driven by the persistence of endemic lineages and P.1 emergence. Nat Med. (2021) 27:1230–8. 10.1038/s41591-021-01378-7 34035535

[15] Kraemer M McCrone J Hill V Bajaj S Evans-Pena R Lambert B. Context-specific emergence and growth of the SARS-CoV-2 Delta variant. medRxiv. (2021) rs.3.rs-1159614. 10.21203/rs.3.rs-1159614/v1 35952712 PMC9534748

[16] Godderis L Luyten J. Challenges and opportunities for occupational health and safety after the COVID-19 lockdowns. Occup Environ Med. (2020) 77:511–2. 10.1136/oemed-2020-106645 32513831

[17] Henriques-Santos BM Farjun B Corrêa IA Figueiredo J de B Fidalgo-Neto AA Kuriyama SN. SARS-CoV-2 variant determination through SNP assays in samples from industry workers from Rio de Janeiro, Brazil. Front Microbiol. (2021) 12:757783. 10.3389/fmicb.2021.757783 35222292 PMC8863740

[18] CDC. Labs. Centers for Disease Control and Prevention. (2020). Available online at: https://www.cdc.gov/coronavirus/2019-ncov/lab/rt-pcr-panel-primer-probes.html (accessed December 28, 2021).

[19] Fintelman-Rodrigues N Silva APD da Santos MC dos Saraiva FB Ferreira MA Gesto J. Genetic evidence and host immune response in persons reinfected with SARS-CoV-2, Brazil. Emerg Infect Dis. 27, 1446–1453. 10.3201/eid2705.204912 33797393 PMC8084520

[20] Cleemput S Dumon W Fonseca V Abdool Karim W Giovanetti M Alcantara LC. Genome detective coronavirus typing tool for rapid identification and characterization of novel coronavirus genomes. Bioinformatics. (2020) 36:3552–5. 10.1093/bioinformatics/btaa145 32108862 PMC7112083

[21] O'Toole Á Scher E Underwood A Jackson B Hill V McCrone JT. Assignment of epidemiological lineages in an emerging pandemic using the pangolin tool. Virus Evol. (2021) 7:veab064. 10.1093/ve/veab064 34527285 PMC8344591

[22] Aksamentov I Roemer C Hodcroft EB Neher RA. Nextclade: clade assignment, mutation calling and quality control for viral genomes. J. Open Source Softw. (2021) 6:3773. 10.21105/joss.03773

[23] Hadfield J Megill C Bell SM Huddleston J Potter B Callender C. Nextstrain: real-time tracking of pathogen evolution. Bioinformatics. (2018) 34:4121–4123. 10.1093/bioinformatics/bty407 29790939 PMC6247931

[24] Sagulenko P Puller V Neher RA. TreeTime: maximum-likelihood phylodynamic analysis. Virus Evol. (2018) 4:vex042. 10.1093/ve/vex042 29340210 PMC5758920

[25] OpenEpi Menu. Available online at: http://www.openepi.com/Menu/OE_Menu.htm (accessed January 18, 2022).

[26] Boletim Epidemiológico N^o^ 77 - Boletim COE Coronavírus — Português (Brasil). Available online at: https://www.gov.br/saude/pt-br/centrais-de-conteudo/publicacoes/boletins/boletins-epidemiologicos/covid-19/2021/boletim_epidemiologico_covid_77-3.pdf/view (accessed January 18, 2022).

[27] Boletim, Epidemiológico. Coronaví*rus*. Available online at: https://coronavirus.rio/boletim-epidemiologico/ (accessed January 18, 2022).

[28] GISAID - Initiative. Available online at: https://www.gisaid.org/ (accessed January 19, 2022).

[29] The The SARS-CoV-2 variant Delta displaced the variants Gamma and Gamma plus in Amazonas Brazil - SARS-CoV-2 coronavirus / nCoV-2019 Genomic Epidemiology - Virological. Available online at: https://virological.org/t/the-sars-cov-2-variant-delta-displaced-the-variants-gamma-and-gamma-plus-in-amazonas-brazil/765 (accessed January 19, 2022).

[30] da Silva Francisco Junior R Lamarca AP de Almeida LGP Cavalcante L Machado DT Martins Y. Turnover of SARS-CoV-2 lineages shaped the pandemic and enabled the emergence of new variants in the state of Rio de Janeiro, Brazil. Viruses. (2021) 13:2013. 10.3390/v13102013 34696443 PMC8537965

[31] Resende PC Delatorre E Gräf T Mir D Motta FC Appolinario LR. Evolutionary dynamics and dissemination pattern of the SARS-CoV-2 Lineage B.1.1.33 during the early pandemic phase in Brazil. Front Microbiol. (2021) 11:615280. 10.3389/fmicb.2020.615280 33679622 PMC7925893

